# Peritonitis caused by *Listeria monocytogenes* and *Burkholderia cepacia* in a patient on peritoneal dialysis: a case report

**DOI:** 10.3389/fmed.2024.1381262

**Published:** 2024-07-17

**Authors:** Yu-Chi Tsai, Ming-Kai Tsai, Wen-Ching Kung, Chien-Yao Wang

**Affiliations:** ^1^Division of Infectious Diseases, Department of Internal Medicine, Kaohsiung Armed Forces General Hospital, Kaohsiung, Taiwan; ^2^Division of Infectious Diseases and Tropical Medicine, Department of Internal Medicine, Tri-Service General Hospital, National Defense Medical Center, Taipei, Taiwan; ^3^Division of Nephrology, Department of Internal Medicine, Kaohsiung Armed Forces General Hospital, Kaohsiung, Taiwan; ^4^Institute of Medical Science and Technology, National Sun Yat-Sen University, Kaohsiung, Taiwan; ^5^Division of General Surgery, Department of Surgery, Kaohsiung Armed Forces General Hospital, Kaohsiung, Taiwan

**Keywords:** case report, *Listeria monocytogenes*, *Burkholderia cepacia*, peritoneal dialysis, peritonitis

## Abstract

Peritoneal dialysis (PD)-associated peritonitis is a major cause of peritoneal dysfunction and failure. The main issue regarding the treatment is whether to remove the catheter surgically or to treat with antibiotics alone. Notably, PD-associated peritonitis is commonly caused by gram-positive cocci, but rarely by *Listeria monocytogenes* and *Burkholderia cepacia*. Here, we report a patient diagnosed with PD-associated peritonitis caused by *L. monocytogenes* and *B. cepacia* who presented with a fever, abdominal pain, and turbid dialysate and had been receiving PD for over 20 years. After 2 weeks of antibiotic treatment, the catheter in the patient was surgically removed. Culture and pathology results revealed pathogen growth, foreign body granuloma with chronic inflammation, and inflammatory cells with fibroblast infiltration. The patient was switched to hemodialysis. She eventually recovered and was discharged. The patient presented fair health at the 3-month follow-up. In conclusion, sequential dialysate white blood cell count may help clinicians decide the course of treatment and guide the timing of surgical intervention.

## 1 Introduction

Peritoneal dialysis (PD)-associated peritonitis is a major cause of peritoneal dysfunction and failure, accounting for 18% of infection-related deaths in patients treated with PD. It is also the primary reason for switching to hemodialysis in patients treated with PD. Patients treated with PD are diagnosed with PD-associated peritonitis based on at least two of the following criteria: abdominal pain and turbid peritoneal effluent, dialysis effluent white cell count >100/μL with more than half being polymorphonuclear cells, and a positive dialysis effluent culture (1).

PD-associated peritonitis is commonly caused by gram-positive cocci, including *Staphylococcus* and *Streptococcus* species, and gram-negative bacilli, including *Escherichia coli*, *Klebsiella* species, and *Pseudomonas aeruginosa* (1). Its treatment mainly involves antibiotic use; surgical removal of the infected PD catheter may be considered in cases of refractory peritonitis, relapsing peritonitis, mycobacterial or fungal peritonitis, or if there is an association with abscess or other intrabdominal lesions (1).

Peritonitis caused by *Listeria monocytogenes* and *Burkholderia cepacia* is rare, and only a few cases have been reported to date (2–6). Consequently, standard evidence-based medical management lacks recommendations for the treatment of this type of peritonitis. Here, we report a patient who was diagnosed with PD-associated refractory peritonitis caused by *L. monocytogenes* and *B. cepacia* and was cured via antibiotic treatment followed by the surgical removal of PD catheter.

## 2 Case description

The patient, a 61-year-old female farmer, was well until 3 days prior to admission, when she developed diarrhea and intermittent, diffuse, and crampy abdominal pain. The patient had been diagnosed with end-stage renal disease due to chronic glomerulonephritis and treated with PD for more than 20 years. Except for hypertension, the patient had no other comorbidities. She was on carvedilol (25 mg bid), amlodipine (5 mg bid), and valsartan (80 mg bid), maintaining a daily blood pressure of 130/60 mmHg. The patient did not smoke, consume alcohol, or use recreational drugs.

The patient ate at a roadside stand with her family 3 days before admission, but denied consuming any raw food. One week before being hospitalized, she temporarily relocated to her farm owing to a demand for overtime work. As the patient only stayed on the farm for a few days, she did not have a proper place to store the peritoneal dialysate. According to the patient and her husband, the farmhouse is often infested with mice. In addition to growing crops, aquaculture of fish, shrimp, and shellfish, and breeding of goose are also practiced on the farm.

The patient’s husband, her main caregiver, was aware that the aseptic principle should be followed when changing the PD solution; however, owing to his time-consuming work and busy schedule, he could not always adhere to good hygiene practices. A week before the onset of her illness, both the patient and her husband noticed that rats often appeared in their house and even found rat feces in the storage space where the PD solution was placed. During the subsequent 2 days, the patient suffered from frequent diarrhea and abdominal pain, which was so severe that it occasionally woke her from sleep. Two days before admission to the hospital, the abdominal pain and diarrhea became even more severe, prompting her husband to take her to the primary care clinic of another hospital.

Upon physical examination, she was noted to have a soft abdomen with low abdominal tenderness. A percutaneous PD catheter was placed in her lower abdomen without peripheral erythematous change. The PD catheter exit site was clean without any discharge. During her visit to the emergency department, the effluent fluid from her catheter appeared turbid. The patient appeared in good physical health and did not have respiratory distress. She presented with the following vital signs: a temperature of 37.9°C, blood pressure of 86/51 mmHg, heart rate of 100 beats/min, and respiratory rate of 18 breaths/min. A computed tomography scan of her abdomen and pelvis revealed a massive amount of intraperitoneal fluid collection with PD catheter retention, without any other space-occupying lesions.

The results of her hemogram indicated leukocytosis with neutrophil predominance and bandemia. The blood C-reactive protein level was also elevated. In addition, the PD effluent appeared white and muddy. The total dialysate leukocyte count of the patient was 2,230 × 10^3^/μL, with 74% neutrophils. The results of the other laboratory tests are shown in Table 1. The patient was admitted to our ward for further management of PD-associated peritonitis.

**Table 1:** Laboratory test results.

Variable	Reference range	Hospital day 1, on presentation	Hospital day 48, on discharge
Hemoglobin (g/dL)	12–15.5	10.6	9.0
Hematocrit (%)	34.9–44.5	31.9	26.5
White-cell count (per mm^3^)	4,000–11,000	39,070	11,960
Differential count (%)			
Neutrophils	40–70	96.0	88.2
Lymphocytes	22–44	2.0	5.1
Monocytes	4–11	1.6	5.3
Basophils	0–3	0.2	0.7
Eosinophils	0–8	0.2	0.7
Band cells	0	12.0	0
Platelet count (×10^3^ per mm^3^)	135–400	326	347
Red-cell count (×10^6^ per mm^3^)	3.90–5.03	3.51	3.00
Mean corpuscular volume (fL)	80.0–100.0	90.9	88.3
C-reactive protein (mg/dL)	<1	20.93	12.92
Lactic acid (mmol/L)	0.5–2.2	1.39	0.43
Sodium (mmol/L)	136–145	133	135
Potassium (mmol/L)	3.5–5.2	2.7	4.8
Urea nitrogen (mg/dL)	8–25	58	
Creatinine (mg/dL)	0.60–1.50	9.3	
Estimated glomerular filtration rate (mL/min/1.73 m^2^)	>60	4.6	
Glucose (mg/dL)	70–110	93	
Aspartate aminotransferase (U/L)	≤33	21	16
Alanine aminotransferase (U/L)	10–49	17	4
Total bilirubin (mg/dL)	0.3–1.2	0.65	0.27
Dialysate effluent sample			
White-cell count (/μL)		2,230	
Red-cell count (/μL)		700	
Differential count (%)			
Neutrophils		74	
Lymphocytes		15	
Monocytes		11	
Dialysate culture	Negative	*Listeria monocytogenes*; *Burkholderia cepacia*	
Dialysate fungal stain and culture	Negative	Negative	
Dialysate acid fasting smear and culture	Negative	Negative	
Dialysate tuberculosis polymerase chain reaction assay	Negative	Negative	
Blood culture	Negative	*Listeria monocytogenes*	

**Figure 1:**
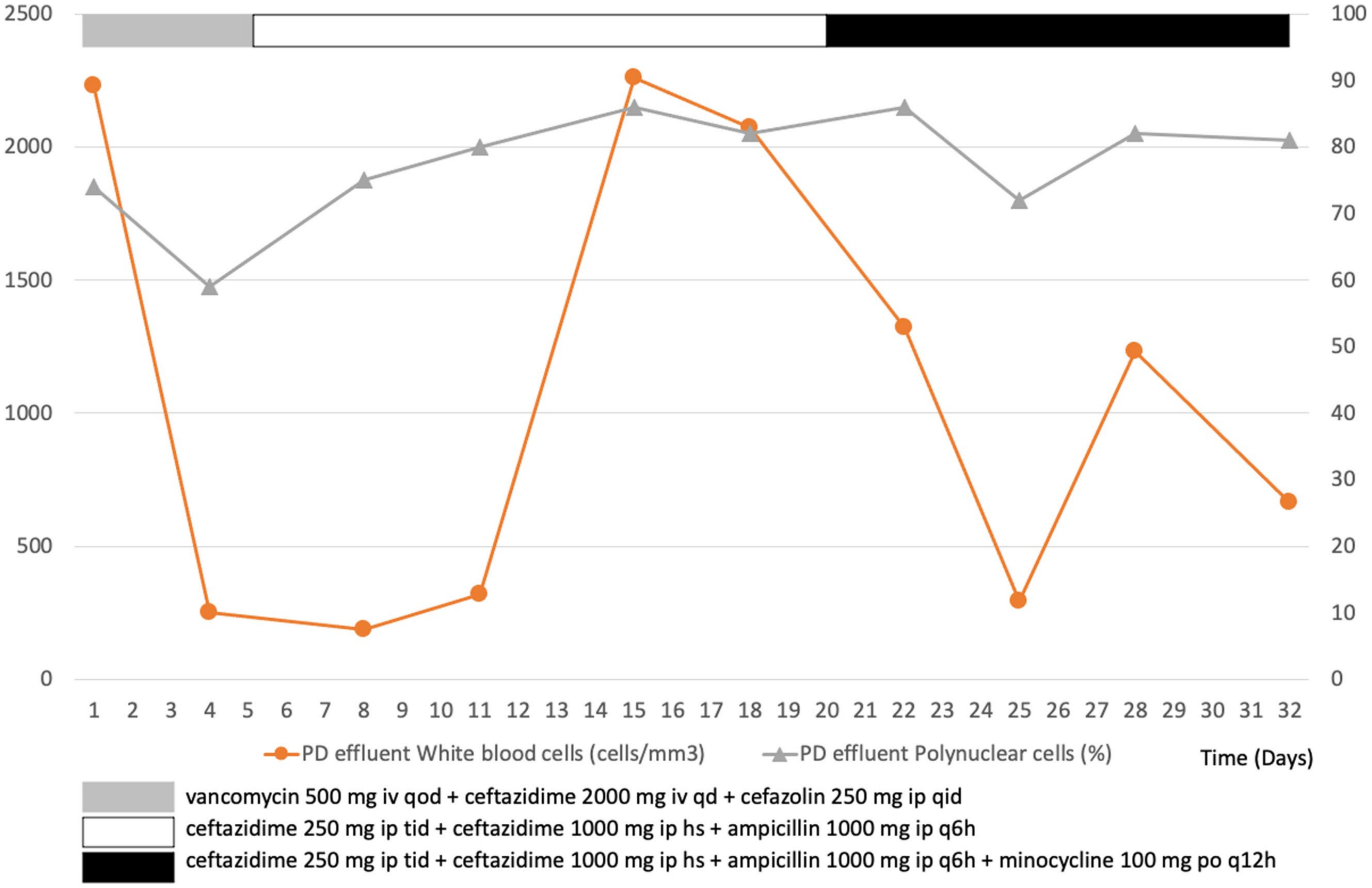
Leukocyte count in peritoneal dialysis (PD) effluent over the course of treatment for a patient with PD-associated peritonitis.

Initially, the patient received empirical treatment with intravenous vancomycin (500 mg qd) and ceftazidime (2 mg qd), as well as intraperitoneal cefazolin (250 mg qid) after obtaining blood and dialysate for culture (Figure 1). On day 3, the blood and dialysate cultures were positive of *L. monocytogenes* and *B. cepacia*. We adjusted the antibiotics to intraperitoneal ceftazidime (250 mg tid plus 1,000 mg hs) and ampicillin (1,000 mg q6h) based on a drug sensitivity test. The patient’s condition improved, with no clinical symptoms of fever, abdominal pain, or turbid dialysate. Although the second follow-up dialysate and blood cultures showed no additional growth of *L. monocytogenes* since day 3, the dialysate culture continued to yield *B. cepacia*. The dialysate leukocyte count also temporarily improved but deteriorated on day 15. Oral minocycline was prescribed on day 20 owing to refractory *B. cepacia* growth, and the patient did not provide consent to the surgical removal of the PD catheter.

On day 34, after 2 weeks of antibiotic treatment, the patient finally consented to the surgical removal of the PD catheter. Severe adhesion of PD cuff to adjacent tissues was observed during the laparoscopic operation. Yellowing of the peritoneum and clean ascites were also observed. The culture and pathology of the removed PD catheter and adjacent tissue showed sterile pathogen growth, foreign body granuloma with chronic inflammation, and inflammatory cells with fibroblast infiltration. A hemodialysis catheter was simultaneously implanted in the patient while removing the PD catheter. The patient was switched to hemodialysis after the surgery. She eventually recovered and was discharged on day 48. The patient presented with a fair health condition at the 3-month follow-up.

## 3 Discussion

*Listeria monocytogenes* is a gram-positive, aerobic or facultative anaerobic, intracellular bacillus that causes listeriosis. Most *Listeria* infections result from foodborne outbreaks, and veterinarians and farmers are at a high risk for listeriosis (2). The most common manifestations of *Listeria* infection are primary bacteremia and meningitis. *Listeria monocytogenes* peritonitis usually occurs in individuals with weakened immune systems and with liver cirrhosis, pregnant women, newborns, and older adults aged >65 years (1, 4). *Listeria monocytogenes* rarely causes peritonitis in patients treated with PD (2). Its clinical presentation is similar to that of the general population, and symptoms include gastroenteritis, fever, meningitis, endocarditis, and secondary or spontaneous peritonitis. However, compared to patients with cirrhosis, those treated with PD have a high survival rate with *L. monocytogenes* peritonitis, possibly because of the early detection of peritonitis symptoms (e.g., abdominal pain and turbid effluent) and regular supervision by health institutions (2, 3). Nervous system infection and septic shock are clinically important symptoms and are indicators of a poor prognosis (3). Penicillin (ampicillin and amoxicillin)-based therapy is the primary regimen for listeriosis. *Listeria monocytogenes* is also susceptible to trimethoprim/sulfamethoxazole, gentamicin, linezolid, and meropenem, and they can be a rationale choice in selected cases (7).

*Burkholderia cepacia* is a ubiquitous, opportunistic, gram-negative bacillus that is present in moist soils, plant rhizospheres, and agricultural products. This bacterium is inherently resistant to multiple antibiotics and is highly transmissible. Treatment for *B. cepacia* infection is generally based on local antimicrobial-susceptibility data. Ceftazidime, meropenem, minocycline, trimethoprim-sulfamethoxazole, and levofloxacin are considered active antimicrobial agents against *B. cepacia* (8). *Burkholderia cepacia* most commonly affects patients with cystic fibrosis and those with compromised immunity and can rarely lead to a PD exit site infection. Most previous cases of *B. cepacia* infection were a part of cluster outbreaks caused by contaminated aqueous chlorhexidine in dialysis wards (9, 10). Fever, abdominal pain, turbid ascites, abdominal distension, nausea, and vomiting are common presentations in the few available case reports (5, 6), along with treatment methods ranging from only antibiotic therapy to the surgical removal of the PD catheter (5). However, comprehensive evidence for an effective treatment method is limited owing to the small number of cases. Notably, *B. cepacia* may form a biofilm on the PD catheter in some cases, leading to the possibility of recurrence (11). Therefore, PD catheters of patients with PD-associated peritonitis exit site infection complicated by tunnel infection should be removed as soon as possible (9, 11).

In this study, *L. monocytogenes* and *B. cepacia-*associated peritonitis may be linked to direct exposure to contaminated water or soil in this patient. The patient’s occupation as a farmer exposes her to an environment where rodents, which could spread *L. monocytogenes*, are commonly present. Damp soil and water sources may also be contaminated with *B. cepacia*. Potential risk factors include poor hygiene in the storage of dialysate and negligence in aseptic techniques during dialysis. Another possibility is contamination from handling food, leading to gastrointestinal infection. However, proving these mechanisms may be challenging.

Most patients with PD-associated peritonitis experience substantial clinical improvement within 48 h of initiating treatment. However, if no improvement is observed within 48 h, the catheter lumen, exit site, tunnel, and dialysate should be reexamined, followed by a dialysate cell count and repeat culture (1).

The clinical significance of dialysate white blood cell (WBC) counts on different days post-antibiotic initiation may vary (12–14). Our patient did not present a dialysate WBC count of <100 cells/μL after 5 days of appropriate antibiotic therapy, meeting the definition of refractory peritonitis despite having a dialysate WBC count of >1,000 cells/μL on day 3 of treatment or a decline rate of <14% on day 5 (1). Therefore, early consideration of surgical intervention to remove her PD catheter was still an option that had to be considered, even though she did not show signs of clinical deterioration based on her vital signs. Moreover, treatment of refractory peritonitis with antibiotics alone without catheter removal is associated with prolonged hospital stay, peritoneal injury, an increased risk of fungal peritonitis, and high mortality (15).

## 4 Conclusion

Overall, PD-associated peritonitis caused by *L. monocytogenes* and *B. cepacia* is rare. Considering the lack of large studies involving a standardized treatment protocol, sequential dialysate WBC counts may help clinicians decide whether antibiotic therapy alone is sufficient or surgical intervention is required.

## Data availability statement

The original contributions presented in the study are included in the article/supplementary material, further inquiries can be directed to the corresponding author.

## Ethics statement

The studies involving humans were approved by Ethics Committee of Kaohsiung Armed Forces General Hospital (approval number: KAFGHIRB 112-014). The studies were conducted in accordance with the local legislation and institutional requirements. The participants provided their written informed consent to participate in this study. Written informed consent was obtained from the individual(s) for the publication of any potentially identifiable images or data included in this article.

## Author contributions

Y-CT: Conceptualization, Data curation, Formal analysis, Investigation, Methodology, Project administration, Resources, Software, Validation, Visualization, Writing – original draft, Writing – review & editing. M-KT: Conceptualization, Data curation, Supervision, Writing – review & editing. W-CK: Conceptualization, Data curation, Supervision, Writing – review & editing. C-YW: Conceptualization, Data curation, Supervision, Writing – review & editing.
